# Assessing the Impact of a Vi-polysaccharide Conjugate Vaccine in Preventing Typhoid Infection Among Bangladeshi Children: A Protocol for a Phase IIIb Trial

**DOI:** 10.1093/cid/ciy1107

**Published:** 2019-03-07

**Authors:** Katherine Theiss-Nyland, Firdausi Qadri, Rachel Colin-Jones, K Zaman, Farhana Khanam, Xinxue Liu, Merryn Voysey, Arifuzzaman Khan, Nazmul Hasan, Fahim Ashher, Yama G Farooq, Andrew J Pollard, John D Clemens

**Affiliations:** 1 Oxford Vaccine Group, Department of Paediatrics, University of Oxford, United Kingdom; 2 International Centre for Diarrhoeal Disease Research–Bangladesh, Dhaka

**Keywords:** typhoid vaccine, cluster randomized controlled trial, protocol, Bangladesh

## Abstract

**Background:**

Typhoid fever illnesses are responsible for more than 100 000 deaths worldwide each year. In Bangladesh, typhoid fever is endemic, with incidence rates between 292–395 per 100 000 people annually. While considerable effort has been made to improve access to clean water and sanitation services in the country, there is still a significant annual typhoid burden, which particularly affects children. A typhoid conjugate vaccine (Vi-TCV) was recently prequalified by the World Health Organization and recommended for use, and offers the potential to greatly reduce the typhoid burden in Bangladesh.

**Methods:**

This study is a double-blind, cluster-randomized, controlled trial of Vi-TCV in a geographically defined area in Dhaka, Bangladesh. At least 32 500 children from 9 months to <16 years of age will be vaccinated and followed for 2 years to assess the effectiveness and safety of Vi-TCV in a real-world setting. All cluster residents will also be followed to measure the indirect effect of Vi-TCV in this community.

**Ethics and Dissemination:**

This protocol has been approved by the International Centre for Diarrhoeal Disease Research, Bangladesh; a University of Oxford research review; and both ethical review committees. Informed written consent and assent will be obtained before enrollment. Vi-TCV has been shown to be safe and effective in previous, smaller-scale studies. The results of this study will be shared through a series of peer-reviewed journal articles. The findings will also be disseminated to the local government, stakeholders within the community, and the population within which the study was conducted.

**Conclusions:**

This trial is the largest and only cluster-randomized control trial of Vi-TCV ever conducted, and will describe the effectiveness of Vi-TCV in an endemic population. The results of this trial may provide important evidence to support the introduction of TCVs in countries with a high burden of typhoid.

**Clinical Trials Registration:**

ISRCTN11643110.

Enteric fever, including typhoid fever, caused by the human-restricted pathogen of *Salmonella enterica,* is responsible for systemic enteric fever illnesses in >20 million people worldwide each year [1]. Of these infections, approximately 100 000–200 000 result in death each year [1]. These infections and deaths largely occur in lower-income countries with poor water and sanitation infrastructures [1]. It has become increasingly apparent that a significant burden of typhoid morbidity and mortality exists in children under the age of 5 years [2–6].

Areas with an incidence of >100/100 000 are considered endemic, including many countries in Africa, South Asia, South-East Asia, and Central Asia [7]. In Bangladesh, studies have estimated an overall population incidence of typhoid between 292–395 per 100 000 people per year [8–10]. These same studies found significantly higher incidences in children under 5 years of age, ranging from 1456–1869 per 100 000 children per year [8–10].

Improvements in infrastructure to provide access to clean water and adequate sanitation and hygiene (WASH), historically, have been the most effective methods for widespread typhoid control. These developments have largely eliminated typhoid as a public health concern from most high-income countries. Between 1995 and 2008, Bangladesh made significant progress in providing improved sanitation services throughout the country [11, 12]. Despite these great efforts, a significant proportion of the poorest individuals are still living without improved sanitation facilities, due to the substantial costs and difficulties in implementing these measures in resource-poor settings [11, 12]. These individuals are often at the greatest risk of typhoid and other enteric infections. As such, the availability and use of an effective vaccine, targeting the highest-risk populations, could be an extremely useful addition to traditional WASH programs.

So far, efforts to control typhoid using vaccination have been limited. In order for a vaccine to be useful as a routine public health tool, it must provide protection in infants and small children, be safe with low reactogenicity, provide long-lasting protection, and be highly effective. The whole-cell typhoid vaccine was reasonably efficacious; however, its high degree of reactogenicity has limited its use [13]. The Vi-polysaccharide vaccine (Vi-PS) overcame reactogenicity issues, but was not licenced for children under 2 years and did not elicit long-term immunological memories, with protection lasting only 2–3 years [14–17]. Developed in the 1980s, the live-attenuated oral vaccine (Ty21a) required 3–4 doses to induce an effective protective immunity and was not licensed for use in children under the age of 6 years, making it a poor candidate for routine use [18, 19]. The Vi-rEPA vaccine—Vi-polysaccharide chemically conjugated to pseudomonas exotoxin A—proved to be highly immunogenic, with a protective efficacy of 91.1% in children aged 2–5 years, and was demonstrated to be immunogenic in infants [20, 21], but has not yet been licensed.

In January of 2018 the World Health Organization (WHO) prequalified a new typhoid conjugate vaccine (Vi-TCV), Typbar-TCV, consisting of 25 µg of Vi-polysaccharide antigen conjugated to a tetanus toxoid carrier protein and manufactured by Bharat Biotech, in India [22]. This vaccine overcomes the barriers limiting previous vaccines: it is safe and immunogenic in infants, children, and adults, and it has the potential to produce long-lasting immunity as a T-dependent vaccine. In preliminary studies, the vaccine showed an efficacy of 54–87% in a human challenge model and has shown a field seroefficacy of 85% (95% confidence interval [CI] 80–88%) [23, 24]. Large-scale field impact studies for Vi-TCV, demonstrating a reduction in the burden of disease attributable to typhoid infections, have not yet been conducted. Following WHO prequalification, and based on expert recommendations for its broader use by the strategic advisory group of experts, Gavi, the Vaccine Alliance has made funding available for countries to introduce Vi-TCV as a typhoid control measure [25, 26].

Currently, TCV is neither licensed nor available for use in Bangladesh. The aim of this study is to assess the impact of Vi-TCV in a real-world field setting, in order to inform and support the use of the vaccine as a control measure for enteric fever in endemic settings. The primary objective is to measure the reduction in blood culture–confirmed typhoid cases in children who receive Vi-TCV, as compared to those who receive a control vaccine. Safety outcomes will also be measured, to increase the knowledge about Vi-TCV tolerability and response in children. By implementing a cluster design, we plan to measure both the direct and indirect effects of Vi-TCV in preventing typhoid infections within the community. Beyond culture-confirmed typhoid cases, the study will measure hospitalizations, abdominal surgical procedures, severe fevers, and missed school and work as the result of fevers for children vaccinated in the cluster populations.

As more countries consider introducing Vi-TCV into routine vaccination programs, understanding the population effects of vaccination will be increasingly valuable. As such, we aim to test Vi-TCV in a real-world, field-impact study.

## METHODS AND ANALYSIS

### Pilot Study

Because Vi-TCV is not yet licensed for use in Bangladesh, prior to implementing the main, cluster-randomized trial, a pilot phase for safety will be conducted in 200 children in an area outside of the main study catchment population. These 200 children will be individually randomized in the pilot phase, in an age-stratified manner, to receive either Vi-TCV or the Japanese encephalitis vaccine (JE). For the pilot phase, 2 sequential groups of children will be studied: children 3–15 years of age (n = 100), followed by children 9 months to <3 years of age (n = 100). Progression from the older age group to the younger age group will be contingent upon a safety review, conducted by the local data safety monitoring board (LDSMB) 1 week post-dosing.

Safety data will be presented to the LDSMB prior to initiating the main, cluster-randomized trial. After this review, and provided approval is given, the main Vi-TCV study will be initiated.

Pilot-study participants will be invited to attend enteric-fever passive-surveillance clinics until the end of the study. For the duration of the 2 years, until unblinding, pilot-study participants will be encouraged to report any serious health events or hospitalizations.

### Study Design

The main study consists of a participant- and observer-blinded, cluster-randomized, controlled trial with a 2-year follow-up to assess the protective impact of the Vi-TCV vaccine. A cluster-randomized design has been selected in order to assess both the individual-level impact (on vaccinees) and population-level impact (on both vaccinees and non-vaccinees, including through indirect protection) of vaccination.

### Population

Children aged 9 months to <16 years who are living in the defined catchment area in Mirpur, Dhaka, and who are in good health at the time of enrollment will be invited to take part. Households with children <16 years of age will be identified and approached for participation by trial staff, including local community health volunteers. At least 32 500 eligible, consenting children/guardians within the target age range (9 months to <16 years) residing in the target area at baseline will be vaccinated.

The participant must satisfy all of the following criteria to be eligible for enrollment:

1. Parent/guardian is willing and competent to provide informed consent. If the participant is 11 to <16 years of age, informed assent will also be sought.
2. Participant is aged between 9 months and <16 years at time of vaccination.
3. Participant is apparently healthy (no complaints of febrile illness) on the day of vaccination.
4. Parent/guardian confirms that their child will be willing and able to comply with study requirements, including follow-up contact, according to the schedule.
5. Participant and parent/guardian live within the study catchment area at the time of vaccination.

The participant will not be enrolled if any of the following criteria apply:

1. Participant has knowingly received a typhoid vaccine or JE in the last 3 years.
2. Participant has a known allergy to any of the vaccine components.
3. There are medical or social reasons that will prevent the participant from conforming to the study requirements, as judged by a medical professional.
4. Participant or parent/guardian is planning to move away from the catchment area within the next month.
5. Participant is pregnant at the time of vaccination, as confirmed by a urine test (urine pregnancy test will be done in girls who are married).

Participants will be temporarily excluded, and asked to return at a later date, if any of the following apply:

1. Participant received any other vaccine in the previous 30 days.
2. Participant has a current temperature of at least 38°C or a reported fever within 24 hours prior to vaccination.
3. Participant used antipyretics within 4 hours prior to vaccination.
4. Participant is a girl between the ages of ≥12 and <16 years old whose first day of her last menstrual period was more than 28 days ago or who does not remember the date of her last menstruation at presentation.

### Study Site

Dhaka, Bangladesh, was selected as the trial site because enteric fever is endemic to Bangladesh, with a documented high incidence in Dhaka. The International Centre for Diarrhoeal Disease Research, Bangladesh (icddr,b) is the local implementing institution for this trial, and has considerable experience conducting high-quality interventional studies. Enteric fever is recognized as a public health concern within the Bangladesh Ministry of Health and the local health authorities in Dhaka. The Ministry of Health is also receptive to impact studies and to the possibility of subsequent vaccination introduction based on those study results. The Strategic Typhoid Alliance across Africa and Asia (STRATAA), funded by Wellcome Trust and the Bill & Melinda Gates Foundation, is a typhoid surveillance study already running in Mirpur, Dhaka [27]. STRATAA has identified a significant burden of disease in the area and a sufficiently large population of children aged 9 months to <16 years within which to conduct this vaccination trial. This age range has been selected because children bear a substantial burden of the disease in both mortality and morbidity, and are likely the source of much of the typhoid transmission within the population at risk.

### Baseline Mapping, Census, and Cluster Formation

The baseline map of the geographically defined catchment area will be created based on satellite images, extracted from Google Earth Pro 7.1.5 and existing Geographic Information System (GIS) Data. The satellite image will be geo-referenced and printed along with existing GIS Data for field mapping and the census. A census field team will visit each structure, road, and other salient geographic feature in the images. They will update geographic features on the satellite image. The census team will also identify all the public and private health facilities, as well as educational and religious institutions, and collect basic information about these facilities.

**Figure 1.**
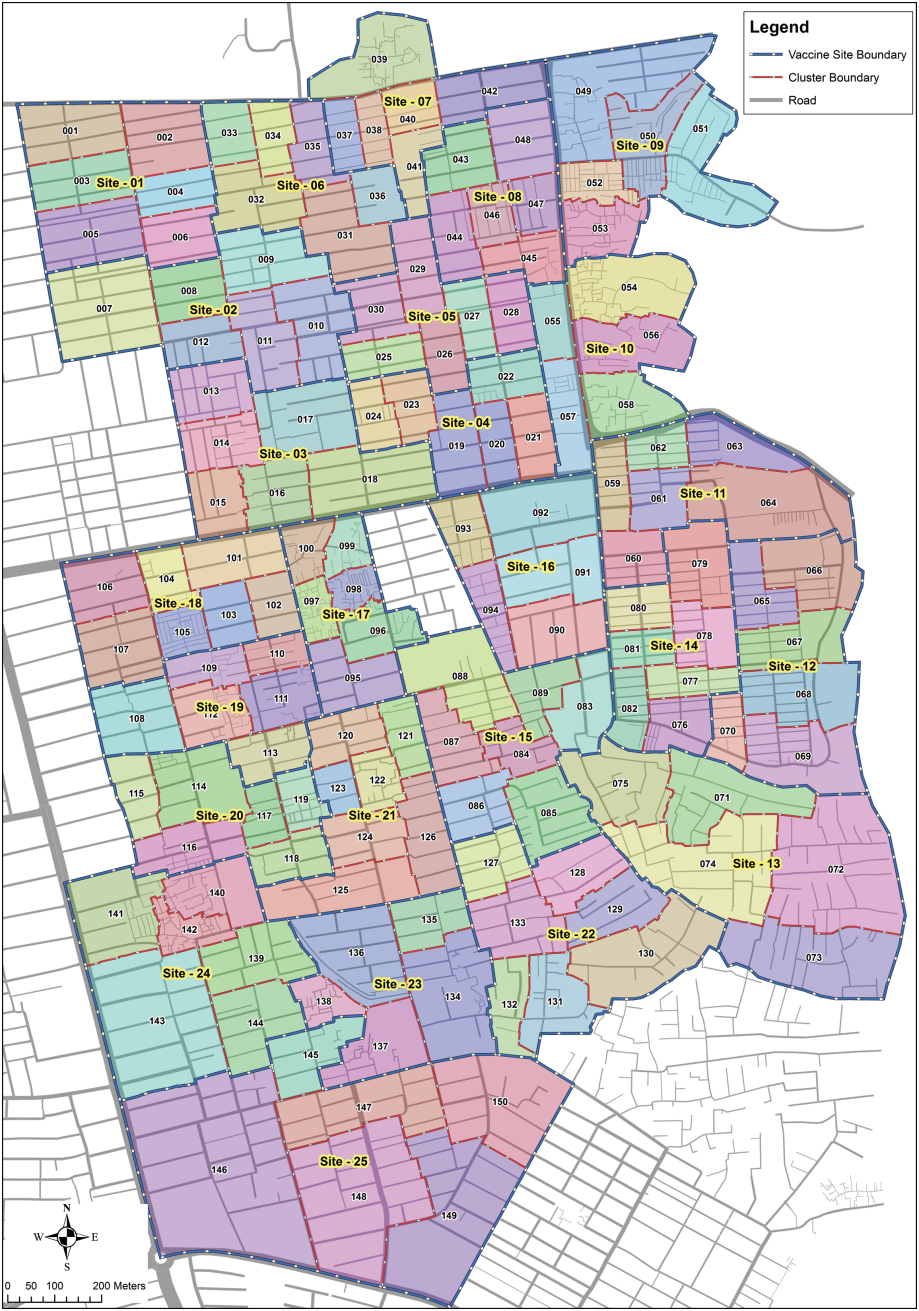
Study area map, indicating 150 cluster and 25 vaccination sites.

A baseline census will enumerate all households within the geographically defined catchment area. Demographic information will be collected on all household members, including, but not limited to: the total size of household, age and sex of all household members, family relationships, and so forth. Verbal consent for the household will be given by either the head of the household or a key informant. After completion, the census population will be merged with GIS data for cluster formation (Figure 1). A census update will be conducted every 6 months for the duration of the trial, to document migration and identify those children eligible for vaccination in the mop-up vaccination campaigns.

The study population for the cluster-randomized control trial will be recruited from a geographically defined area of Mirpur, Dhaka, Bangladesh. The study area will be divided into 150 clusters. Each cluster will consist of a total population of approximately 1250 people. Clusters will be demarcated by road networks and will include landmarks and physical features. Others areas, such as commercial places, education zones, and playgrounds, will also be considered for cluster demarcation. The cluster margins will be adjusted so that they are aligned with natural divisions to separate residences in the community. An inner cluster line will be defined to exclude a population of approximately 200–300 people at the boundaries of each cluster, which will support in separating 2 adjacent clusters to minimize the contamination between clusters. The entire cluster population (not just the inner cluster) will be eligible for vaccination. The inner cluster will include a population size of approximately 950–1050 people and will serve as the population under primary analysis, though the full cluster will be analyzed in secondary analyses.

Clusters will be randomized in a 1:1 ratio to either the Vi-TCV arm or the JE arm of the study. A pre-prepared randomization list will be uploaded into a purpose-built, offline, mobile application. This application will be used at the first study visit to identify which vaccine has been randomized to the cluster in which the child lives. Login details that allow access to the application will only be given to unblinded staff. No trial staff will be able to make changes to the randomization information stored on the application.

### Intervention

**Figure 2.**
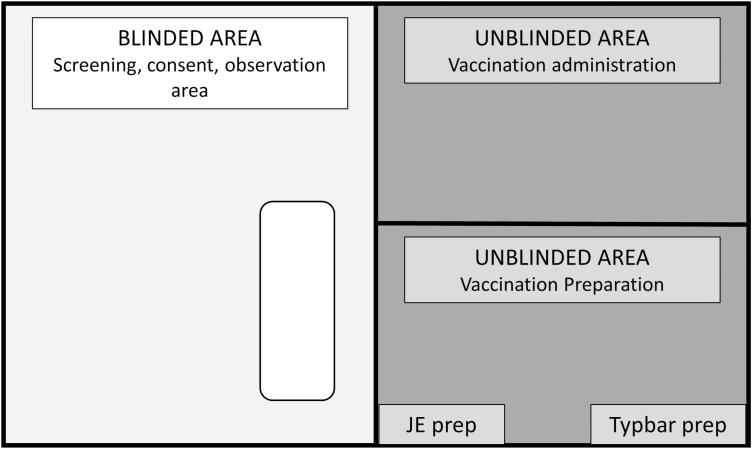
Diagram of blinded and unblinded areas at vaccination site. Abbreviations: JE, Japanese encephalitis vaccine; Typbar, a typhoid conjugate vaccine.

Children enrolled in the study will present at 1 of 25 vaccination sites set up to deliver the vaccination campaign at the start of this study. Each child will receive either the Vi-TCV or JE vaccine, based on which cluster they live in at the time of study enrollment. The JE vaccine has been selected as the control vaccine because Japanese encephalitis is endemic to Bangladesh, and so the vaccine provides a public health benefit to children randomized to control clusters. In order to maintain blinding, use of the randomization application and vaccination preparation will take place behind a curtain. Only unblinded staff will be permitted to be in this area. Vaccination administration will happen in an area that is curtained off from both the vaccination preparation and the blinded area. Consent and screening will take place in the blinded area (Figure 2). Follow-up contacts will not be conducted by unblinded staff.

A subset of approximately 4800 participants will be selected on a 1:1 basis (Vi-TCV vs JE) from all 150 clusters to be contacted by telephone or in person 7 days after vaccination, for follow-up and to record any adverse events following vaccination. The selection of these participants will be age-stratified (<5 years vs ≥5 years of age, with an allocation ratio of 1:1). All participants will be able to access a trial doctor to report any adverse events post-vaccination.

A subset of approximately 1500 participants will be selected on a 2:1 basis (Vi-TCV vs JE) to have blood samples collected at baseline (Day [D] 0), at D28, at 18 months (D545), and at 2 years (D730) post-vaccination to study immunogenicity. The selection of these participants will be age-stratified (<5 years vs ≥5 years of age, with an allocation ratio of 1:1).

**Figure 3.**
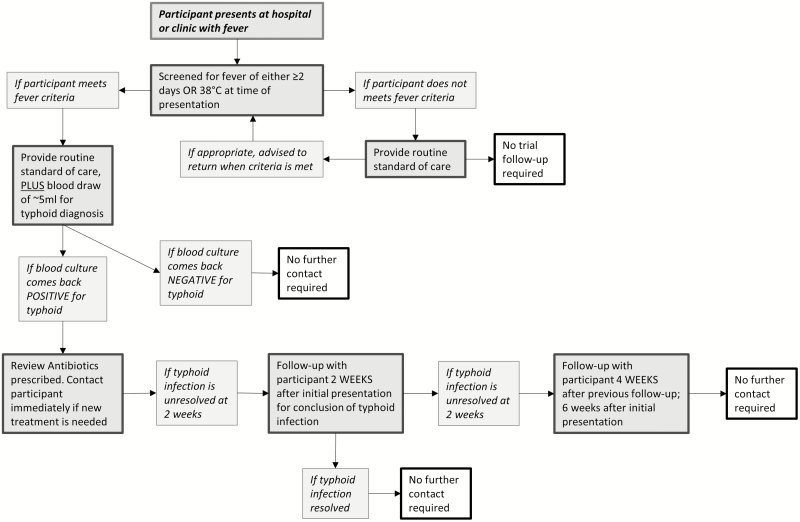
Unscheduled presentation with fever, in a flow chart.

Surveillance for enteric fever will be undertaken in at least 9 health-care facilities in Mirpur, for all residents of the participating clusters, for a minimum of 1 month preceding baseline and continuing for approximately 2 years after vaccination, until the end of the study. In this surveillance, all consenting residents from the participating clusters who present with a subjective history of ≥2 days fever and/or a temperature of ≥38°C will have a blood culture taken (6–10 ml, depending on age), and will receive appropriate clinical management. Those with positive blood cultures will be visited at home to confirm their identity, as given at the treatment center; to collect information about their illness; and to review the treatment given and adjust as necessary, based on laboratory testing (Figure 3).

Participants who present to passive-surveillance health-care facilities with symptoms of Japanese encephalitis infections will each have a blood sample collected, which will be tested for antibodies using an enzyme-linked immunosorbent assay (ELISA) kit.

At 6-month intervals during the 2 years after baseline, census updates of the population will be done in all clusters. At the time of the census updates, information about all deaths, births, and hospitalizations since the previous survey will be collected. At these updates, vaccinated participants’ parents/guardians will also be queried about surgeries, serious adverse events, and other significant illnesses occurring since the last census. Verbal autopsies will be done for the deceased among all cluster residents.

At 6-month intervals, mop-up vaccination campaigns will take place to vaccinate those children who have aged into the study, migrated into the area, or were missed in previous campaigns, in order to maintain vaccination coverage in each cluster for the duration of the study. In these mop-up campaigns, children will be vaccinated with the vaccine allocated to the cluster in which they reside. Final vaccination will be done at 18 months. A complete schedule of planned procedures can be seen in Table 1.

**Table 1.** Visit and Sample Schedule

Visit	1	2	3	4	5	6	7	Trial Completion^a^
Days post-vaccination	0	7	28	180	365	545	730	730 post–trial start^a^
Permissible time window, in days	…	+7/-1	±4	±28	±56	±56	±90	±90
Screening	X	…	…	…	…	…	…	…
Consent	X	…	…	…	…	…	…	…
Randomization	X	…	…	…	…	…	…	…
Vaccination	X	…	…	…	…	…	…	…
Medical history and exam	X	…	…	…	…	…	…	…
Blood collection^b^	X^c^	…	X	…	…	X	…	X
Blood volume	~3–5 ml	…	~3–5 ml	…	…	~3–5 ml	…	~3–5 ml
AEFI follow-up^d^	…	X	…	…	…	…	…	…
Census update and follow-up contact^e^	…	…	…	X	X	X	X	…
Unblinding and documentation of vaccination	…	…	…	…	…	…	…	X

Abbreviation: AEFI, adverse event following immunisation.

^a^All participants will be unblinded at the same time, at ~730 days after the first participant is enrolled, regardless of when an individual joined the study. Participants who were enrolled during a census update will only complete those visits occurring between their enrollment and the subsequent completion of the trial.

^b^The blood collection will be in a subset of 1000 Vi-typhoid conjugate vaccine and 500 control participants. The total volume will be ~12–20 ml per participant.

^c^The blood draw on Day 0 will occur before vaccination.

^d^At Day 7, a subset of participants will be contacted to collect all AEFIs.

^e^Follow-up contact includes: compiling a census update and ensuring the participant and family still live in the area and are happy to continue with the study; recording mortality and morbidity in participants, including serious adverse events; and providing a reminder to attend the trial health-care facility if the participant develops a fever of ≥2 days.

At 2 years after the initial vaccination campaign, the trial will end and all participants and trial staff will be unblinded. At this point, pilot-study participants and both the control and intervention groups of the main trial will be informed of their vaccination statuses and have their vaccines documented on their health records. All participants in the control group will then be offered vaccination with the Vi-TCV vaccine.

The duration of participation is up to 2 years from enrollment (shorter than 2 years for subjects enrolled and vaccinated during the post-baseline, 6-month mop-up vaccination campaigns).

### Outcomes

The primary outcome of interest is blood culture–confirmed typhoid in children vaccinated with the intervention vaccine, as compared with the control vaccine.

Secondary outcomes will be determined by following both the vaccinees and all cluster residents and will include: hospitalizations, abdominal surgeries, severe fevers and typhoid cases, absences from work and/or school as the result of illnesses, and mortality.

These outcomes will be measured through passive surveillance at study fever clinics and through participant follow-up contacts. Trial participants and cluster residents who report at least ≥2 days of subjective, persistent fever and/or present with a temperature of at least 38°Cwill be tested for typhoid via blood culture. Positive cases will be treated with antibiotics and followed until resolved. Participants and cluster residents will also be contacted every 6 months to collect self-reported health information (Table 1).

The blood samples collected from participants enrolled into the immunogenicity sub-study will be used to investigate the duration and persistence of anti-Vi antibodies over time. IgG Vi antibodies will be measured using the established, commercial ELISA vacyme and the WHO-approved serum standard. Blood samples collected from suspected typhoid cases will have DNA extracted for host genetics studies. DNA will be whole-genome genotyped using Illumina genotyping arrays. Genome-wide data will be used for quantitative trait locus analysis, to identify those genes that contribute to the Vi antibody response.

### Sample Size

Sample size calculations are based on the following assumptions:

1. An overall incidence of typhoid fever of 50 cases per year per 100 000 persons in the entire population, with higher incidence rates in children under 16 years.
2. Age-specific incidence rates were determined from the age distribution of typhoid cases from earlier cohort studies in Bangladesh.
3. A direct protective effect of vaccination of 80% (protection of vaccinees) and an indirect effect of 20% (protection of neighboring non-vaccinees), as predicted from mathematical modelling.
4. A coefficient of variation of .5.
5. Cluster size (of the inner clusters) of 1000, of whom an expected 289 will be aged 9 months to <16 years.
6. A 60% average vaccine coverage of the target age group throughout follow-up.
7. A total 2 years of follow-up.

Based on the above assumptions, the sample size calculated for use in this trial will be 43 350 children within 150 clusters, with 1000 participants on average in each inner cluster, and with all participants randomized 1:1 to receive either the Vi-TCV or JE vaccine. The total number of children eligible for vaccination in both the inner and outer clusters is, therefore, 43 350 × 1.25 = 54 188, of which we expect approximately 60% to participate, resulting in approximately 32 500 children vaccinated.

With these assumptions, the trial will have 98% power to detect a 82% level of total vaccine protection by Vi-TCV and 74% power to detect a 43% level of overall protection by Vi-TCV and a *P* value < .05 (2-tailed).

### Data Management

Electronic devices and hard copies of case report forms (CRFs) will be used to collect and record all data in trial CRFs. CRF and randomization data will be collected offline and uploaded to a secure server on a regular basis, when electronic devices are brought back to the central field office and reliable Internet is available. Some CRFs will also be completed online.

Census data will be collected on an electronic device with an Android application in the open data kit (ODK) system, developed by Bangladesh information technology and data management teams. All other CRFs will be designed and maintained on Research Electronic Data Capture (REDCap), a secure web application for building and managing online surveys and databases. Both the ODK and REDCap systems will be validated by data management and information technology staff within Oxford University. Separate CRFs will be designed as per the necessity of different components of the study, will be maintained by a dedicated trial data manager, and will have quality control checks performed on a regular basis. All participants will be identified by a unique, trial-specific number and/or code; this will not include any identifiable information. Each trial staff member will have an appropriate level of access to the CRFs and collected data, according to their role and responsibilities within the trial.

All participant data will be stored and maintained on local servers in Bangladesh for the duration of the trial. Anonymized data will be shared with Oxford University in the United Kingdom. At the end of the trial, all individually identifiably data will be removed. For the purpose of analysis, fully anonymized data will be retained electronically until the youngest child participating in the trial reaches 21 or for 5 years following completion of the research, whichever is longer.

### Data Analysis Plan

The primary endpoint will be blood culture–positive typhoid fever, detected in passive surveillance at the study surveillance sites (with passive surveillance undertaken for persons residing in the study area) and in the follow-up visit of all vaccinees each 6 months.

We will test the hypothesis that there is no difference in the incidences of blood culture–confirmed typhoid fever between Vi-TCV and the control vaccine group, using a survival analysis and adjusting for design effect and prespecified prognostic factors. The design effect of the cluster randomization will be adjusted as random effects, using a shared frailty model or robust sandwich variance estimates where the shared frailty model cannot be converged. Kaplan-Meier survival curves will also be presented.

Vaccine efficacy will be calculated as (1 − HR) × 100%, where HR is the hazard ratio (Vi-TCV: control) from the random-effects proportional-hazards model. Where the proportional-hazards assumption is not met, the vaccine efficacy will be calculated based on the relative risk, estimated by appropriate statistical analyses.

Participants will be censored in the analysis at the time of their last known residence in the surveillance area, death, or at the 2-year final visit. Statistical significance will be determined as a *P* value of less than .05 (2-tailed). The primary analyses will address total vaccine protection (comparing the incidence of typhoid in vaccinees in the 2 arms) and the secondary analyses will address overall protection (comparing the incidence of typhoid in the 2 arms, regardless of whether the vaccine assigned to the cluster was received). In the secondary analyses, we will also estimate indirect vaccine protection (comparing the incidence of typhoid in non-vaccinees in the 2 arms).

The proportional hazards assumption will be verified for all independent variables before conducting the Cox proportional hazards regression analysis. If the assumption is not met, an appropriate survival analysis or other regression technique will be applied. The details of model selection under different scenarios will be specified in the statistical analysis plan.

 To evaluate the heterogeneity of Vi-TCV vaccine protection among different subgroups, we will evaluate interaction terms between the vaccination and subgroup variables in these models. The *P* values for these analyses will be calculated as 2-tailed. The study is not powered to detect differences between sub-groups, and any observed patterns will be interpreted cautiously, owing to the large study population and increased chance of a Type I error.

## OTHER ETHICS CONSIDERATIONS

Written and verbal versions of the Participant Information and Informed Consent/Assent form will be presented to the participants’ parent/guardian in the local language, detailing no less than: the exact nature of the trial; what it will involve for the participant; the implications and constraints of the protocol; the known side effects; and any risks involved in taking part. It will be clearly stated that the participants’ parent/guardian is free to withdraw their child from the trial at any time, for any reason, without prejudice to future care, without affecting their legal rights, and with no obligation to give the reason for withdrawal.

This study will be monitored by both a local and an international DSMB. The LDSMB and DSMBs will be independent of the study team and will provide safety monitoring oversight for the duration of the study. Reports on the trial progress, protocol deviations, and safety outcomes will be presented to the DSMBs at regular intervals for review. The DSMB has the power to halt the trial, under certain conditions, if there is a concern about participant safety.

The Investigator will ensure that this trial is conducted in accordance with the principles of the Declaration of Helsinki, relevant regulations, and the principles of Good Clinical Practice. The study will be conducted based on the ethical requirements of both icddr,b and the University of Oxford. This protocol, and all participant-facing documents, have been reviewed and approved by the research review and ethics review committees at icddr,b and the University of Oxford Tropical Research Ethics Council.

## CONCLUSION

This trial will measure the effectiveness of the Vi-TCV vaccine in a population setting, after vaccination of children with either Vi-TCV or a JE control vaccine. This will be the largest trial of Vi-TCV to date and the only cluster-randomized control trial, which may be able to measure both direct and indirect effects of the vaccine. Generating this type of evidence may support countries with endemic typhoid in their decision-making processes when considering introducing Vi-TCV as a routine childhood vaccine.

This protocol details the design and implementation of a large-scale, cluster-randomized control trial in a lower-income country setting. These types of trials are extremely important to understand the impact of new vaccines in real-world settings and endemic areas. This protocol can support other research efforts to design and implement similar trials of this nature.

## Notes

***Author contributions.*** K. T.-K., F. Q., and R. C.-J. developed the research protocol. A. J. P. and J. D. C. provided the master plan leading to the research framework, contributed to the protocol development, and provided advice on research oversight. F. K. supported the clinical delivery plan and protocol finalization. X. L. and M. V. wrote the analysis plan and statistical evaluation for the sample size. K. Z., A. K., N. H., F. A., and Y. G. F. supported the protocol finalization.

***Acknowledgments.*** The authors thank the Bill & Melinda Gates Foundation for funding the Typhoid Vaccine Acceleration Consortium, including this trial (grant number OPP1151153); the Wellcome Trust and the Bill & Melinda Gates Foundation for funding The Strategic Typhoid Alliance Across Africa and Asia, which supported the surveillance that underpins this trial; and the National Institute for Health Research Oxford Biomedical Research Centre.

***Disclaimer.*** The views expressed in this article are those of the author(s) and not necessarily those of the Joint Committee on Vaccination and Immunisation, National Health Service, the National Institute for Health Research, the World Health Organization, or the Department of Health. The findings and conclusions contained within are those of the authors and do not necessarily reflect the positions or policies of the Bill & Melinda Gates Foundation.

***Potential conflicts of interest.*** A. J. P. is a National Institute for Health Research Senior Investigator; chairs the UK Department of Health and Social Care’s Joint Committee on Vaccination and Immunisation; is a member of the World Health Organization’s Strategic Advisory Group of Experts; and has received grants from Pfizer and Okairos, outside the submitted work. All other authors report no potential conflicts. All authors have submitted the ICMJE Form for Disclosure of Potential Conflicts of Interest. Conflicts that the editors consider relevant to the content of the manuscript have been disclosed.

***Financial support.*** This publication is based on research funded in part by a grant from the Bill & Melinda Gates Foundation (OPP1151153).

***Supplement sponsorship.*** This supplement is sponsored by the Center for Vaccine Development and Global Health (CVD) at the University of Maryland School of Medicine.
